# Gastric metastasis arising from invasive ductal and mucinous carcinoma of the breast: a case report and literature review

**DOI:** 10.3389/fonc.2025.1576874

**Published:** 2025-08-21

**Authors:** Zhidong Lyu, Jie Fei, Longxiao Zhang, Yinbo Liu

**Affiliations:** ^1^ Department of Breast Surgery, The Affiliated Hospital of Qingdao University, Qingdao, China; ^2^ Pathology, The Affiliated Hospital of Qingdao University, Qingdao, China; ^3^ Information Technology Management, The Affiliated Hospital of Qingdao University, Qingdao, China

**Keywords:** breast cancer, gastric metastasis, HER-2, CDK4/6 inhibitor, case report

## Abstract

Gastric metastasis of breast cancer is rare, and clinical data on its treatment and prognosis are limited at present. Herein, we report a case of gastric metastasis arising from invasive ductal and mucinous carcinoma of the breast and review the literature. A 51-year-old woman was diagnosed with infiltrating and mucinous carcinoma of the right breast accompanied by ipsilateral axillary lymph node and subclavian lymph node metastases. The molecular subtype was luminal A, and the clinical stage was T4bN3M0. The patient first received 5 cycles of neoadjuvant chemotherapy, and the treatment efficacy was stable. After 3 months of treatment with abemaciclib combined with exemestane, the tumors did not significantly reduce in size. The patient underwent surgery on February 23, 2023. The postoperative pathological examination revealed Miller Payne system grade 3 (G3). Cancer metastasis (14/20) was observed in the axillary lymph nodes, and the immunohistochemical results were as follows: ER (++, 90%), PR (+, 2%), HER-2 (3+), and Ki-67 (20%). The adjuvant therapy used was exemestane combined with trastuzumab and pertuzumab. Gastric metastasis was observed 10 months after surgery. The pathological examination revealed focal atypical cell nests with disordered arrangements, indicating malignant lesions (cancer). The immunohistochemical results were as follows: GATA3 (+), ER (++, 90%), PR (-), and HER-2 (1+). In the late stage, first-line treatment with dalpiciclib and fulvestrant was administered, and liver metastasis occurred 10 months thereafter. T-DXd treatment was subsequently administered, and the patient is currently undergoing clinical follow-up. This case highlights the possibility of gastric metastasis when gastrointestinal symptoms occur in patients with a history of breast cancer.

## Introduction

Breast cancer is a common disease that is a cause of considerable concern for women worldwide (1). Common metastatic sites for breast cancer include the lymph nodes, bones, lungs, and liver, whereas the gastrointestinal tract is a rare site for breast cancer metastasis and is often overlooked by clinicians. Retrospective clinical analysis revealed that the proportion of patients with breast cancer metastasizing to the stomach ranges from 0.1% - 0.3%. Previous research has indicated that 97% of gastrointestinal metastases originate from invasive lobular carcinoma (2). Reports of gastric metastasis of invasive ductal carcinoma are relatively rare. Mucinous adenocarcinoma of the breast is a special type of breast cancer that accounts for 1.4% - 5.2% of all breast cancer cases and is typically characterized by slow tumor growth, infrequent metastasis, and a better prognosis than other types of breast cancer (3).

In this article, we report a rare case of invasive ductal and mucinous carcinoma of the breast presenting with gastric metastasis. The symptoms of gastric metastasis differ from those of breast cancer, with clinical symptoms primarily including belching, acid reflux, poor appetite, and other types of upper abdominal discomfort. Endoscopic findings of gastric metastasis are similar to those of primary gastric cancer. Under endoscopy, it can present as isolated protruding lesions, submucosal lesions, gastric wall ulcers, etc. (4). Herein, we report a case and review the literature to increase the degree of clinical awareness of gastric metastasis of breast cancer.

## Case presentation

**Figure 1:**
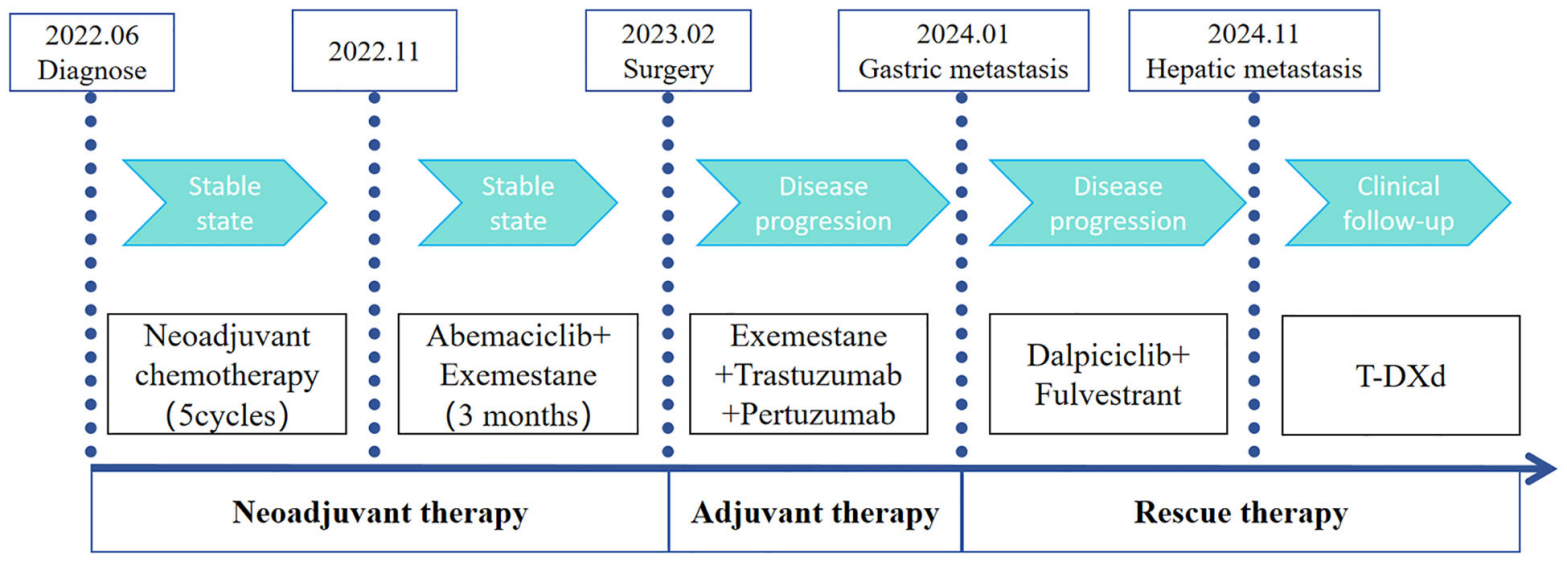
The schedule of treatment process.

A 51-year-old woman was diagnosed with invasive carcinoma on June 13, 2022. Physical examination revealed that the right breast was significantly larger than the left, with a palpable mass in the right breast measuring 10.0 cm × 10.0 cm. The skin over the mass was red and swollen, and the mass was hard, ill-defined, and irregular in shape. Multiple enlarged lymph nodes were palpable in the right axilla. Her Eastern Cooperative Oncology Group (ECOG) status was 0, and her treatment is summarized in **Figure 1**. Ultrasound of the breast revealed a mixed cystic and solid mass in the right breast that was 10.0 cm × 9.0 cm in size. Multiple enlarged lymph nodes were observed in the right axilla and subclavicular region. The Breast Imaging Reporting and Data System (BI-RADS) category was 5. Mammography of the breast revealed a calcified mass in the right breast (BI-RADS 5), and the lymph nodes in the right axilla were enlarged and dense. Core needle biopsy pathology showed invasive carcinoma (histological grade II), mostly mucinous carcinoma. (Right axillary and subclavicular lymph node) Cancer metastasis within lymphoid tissue. The immunohistochemical (IHC) results revealed the following: ER (+++, 7%), PR (+++, 60%), CerbB-2 (1+), and Ki-67 (10%). The clinical stage was cT4bN3M0. No significant abnormalities were found in the whole-body examinations.

**Figure 2:**
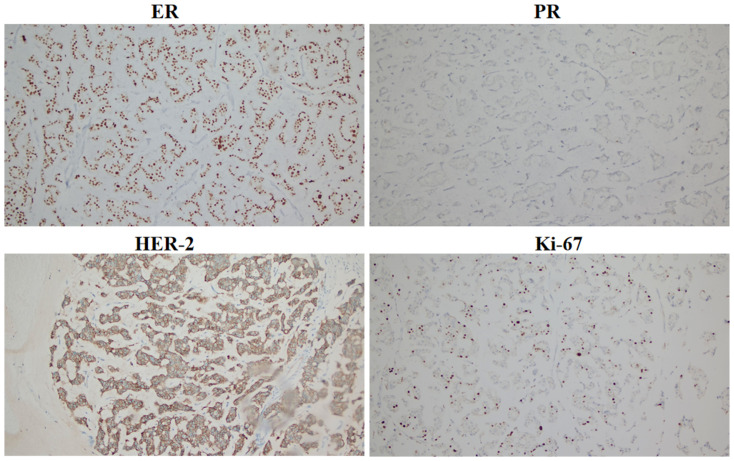
Immunohistochemical expression of ER, PR, HER-2, and Ki-67 in breast cancer after surgery (100**×**).

The patient had locally advanced breast cancer and was given neoadjuvant chemotherapy comprising 0.9 g of cyclophoshamide, 130 mg of epirubicin, and 400 mg of albumin-bound paclitaxol every 21 days. After the fifth cycle of chemotherapy, MRI revealed that the breast lumps and lymph nodes were similar to before. Endocrine therapy was considered a suitable treatment. Starting on November 5, 2022, the patient received cyclin-dependent kinase 4/6 (CDK4/6) inhibitors in combination with endocrine therapy comprising abemaciclib (150 mg) and exemestane (25 mg). After 3 months, her disease remained stable. The patient underwent a modified radical mastectomy with flap transfer and reconstruction (locally based back flap rotation) on February 23, 2023. Pathology revealed cystic changes in partially excised breast tissue, with residual mucinous carcinoma (histological grade II, maximum range 3.0 cm × 2.8 cm) near the cystic area. The Miller–Payne grading system was grade 3 (G3). Metastasis of cancer was found in the lymph nodes of the ipsilateral axilla (14/20). The IHC results were as follows: ER (+++, 9%), PR (+, 2%), CerbB-2 (3+), and Ki-67 (20%) (**Figure 2**). On the basis of the results of the APHNINITY study, the patient received endocrine therapy combined with targeted therapy comprising exemestane (25 mg), trastuzumab (initial dose 8mg/kg, subsequent 6mg/kg), pertuzumab (initial dose 840 mg/kg, subsequent 420 mg/kg). In June 2023, the patient started radiation therapy of the chest wall and regional nodes (50 Gy in 25 fractions) and subsequently underwent regular follow-up examinations.

**Figure 3:**
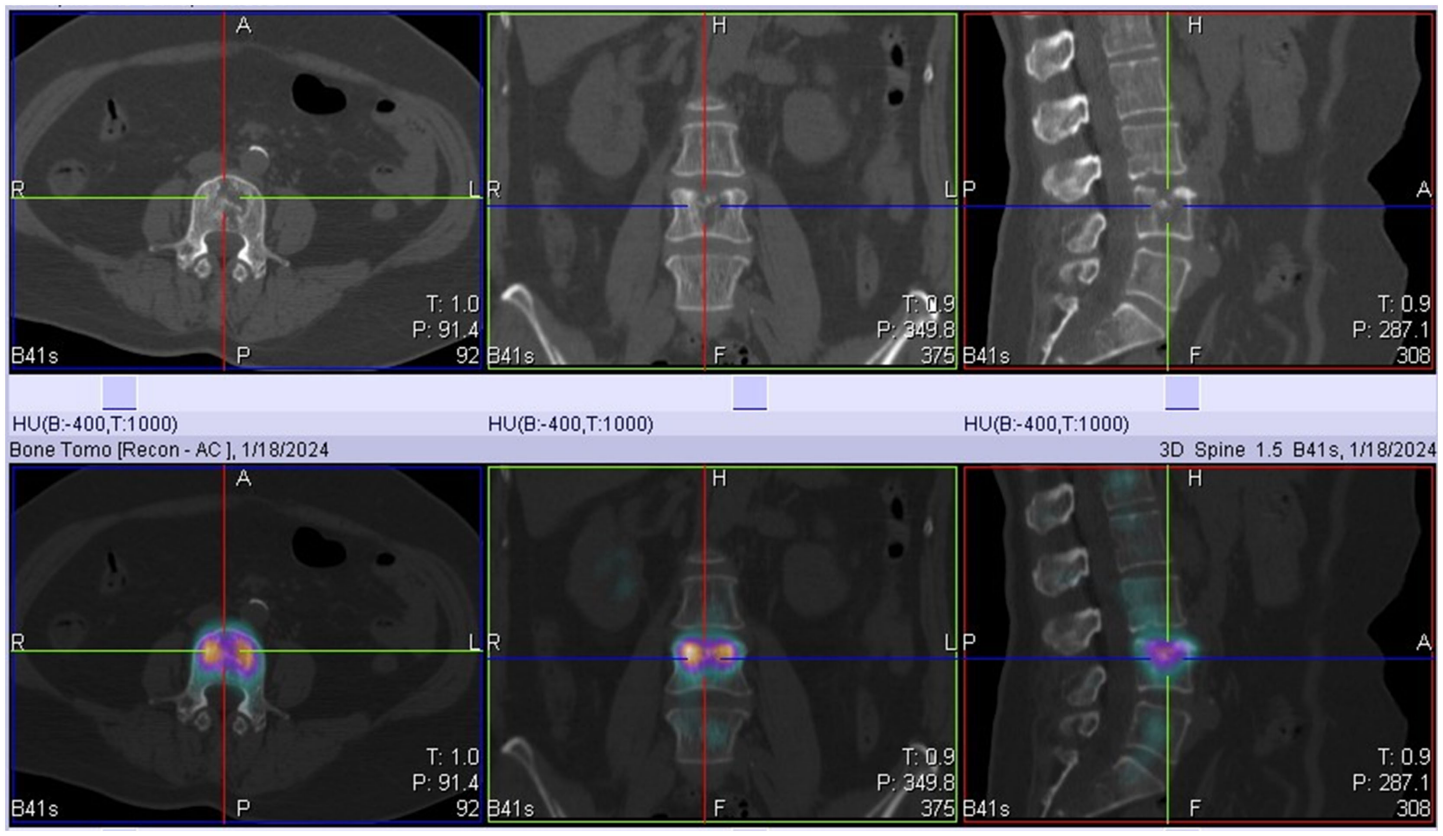
Whole-body bone scan suggests the 4th lumbar spine destruction.

**Figure 4:**
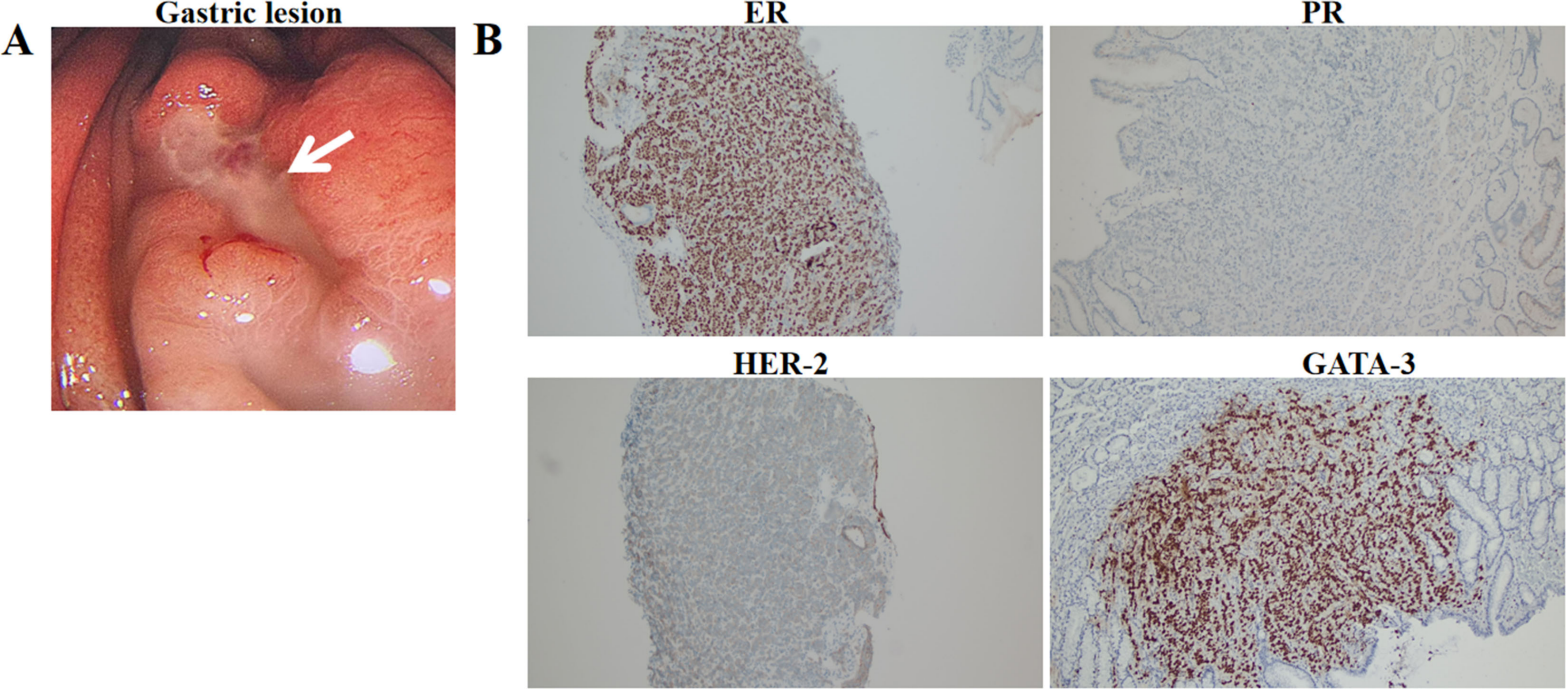
Gastric lesion under gastroscopy **(A)** and immunohistochemical expression of ER, PR, HER-2, GATA-3 in gastric lesion (100**×**) **(B)**.

On January 18, 2024, SPECT/CT revealed multiple abnormal foci of concentrated imaging agent in the 9th thoracic vertebra, 4th lumbar vertebra, and left ilium (**Figure 3**). The patient exhibited symptoms of vomiting blood, and gastroscopy revealed a deep ulcer with a diameter of 10 mm on the anterior wall of the stomach. The pathological results revealed focal nest-like atypical cells with disordered arrangements, indicating malignant lesions. The IHC results revealed the following: GATA3 (+), ER (+++, 9%), PR (-), and CerbB-2 (1+) (**Figure 4**). Combined with the morphological and immunohistochemical findings, these findings confirmed the presence of breast cancer metastasis. On the basis of the results of the DAWNA-1 trial, dalpiciclib (150 mg) and fulvestrant (0.5 g) were used as first-line treatments. After 10 months, CT revealed circular low-density areas with unclear boundaries in the right posterior lobe and left medial lobe of the liver (21 mm × 18 mm). Liver nodule biopsy revealed cancer infiltration in the liver tissue. The IHC results were as follows: GATA3 (+), ER (++, 80%), PR (-), CerbB-2 (2+), Ki-67 (20%), hepatocyte (-), and HER-2: negative (no amplification). On the basis of the results of the DESTINY-Breast 4 study, T-DXd treatment was administered (5.4 mg/kg every 21 days) as second line of treatment began on November 20, 2024. The patient is currently under clinical follow-up.

## Discussion

This article describes a rare case of breast cancer metastasis to the stomach, bones, and liver after surgery. It is commonly believed that gastric metastasis is most likely to occur within 5 to 7 years following the initial diagnosis of breast cancer (5). Invasive lobular carcinoma has a greater potential to metastasize to the gastrointestinal tract than invasive ductal carcinoma does. Taal et al. reported 51 cases of metastatic breast cancer diagnosed through gastroscopy, including 36 cases of invasive lobular carcinoma and 10 cases of invasive ductal carcinoma, with the remaining 5 cases being other types of tumors (6). Another retrospective study revealed that, out of 35 patients with gastric metastasis, 34 patients had invasive lobular carcinoma of the breast, suggesting that invasive lobular carcinoma is more likely to metastasize to the stomach (7). Studies have revealed that a lack of E-cadherin leads to an irregular and aggressive morphology of invasive lobular carcinoma, resulting in a loss of adhesive properties and a gain in cell mobility, thereby improving invasiveness (8). In addition, invasive lobular carcinoma may produce a signet ring morphology, which is easily confused with primary gastric carcinoma (9). In this article, we describe a case of gastric metastasis after surgery for invasive ductal and mucinous carcinoma of the breast, indicating that gastric metastasis of invasive ductal carcinoma of the breast also requires attention. Gastric metastases of breast cancer are difficult to distinguish from primary gastric cancer (8). Therefore, a combination of biopsy, pathology, and endoscopy is needed, as is a differential diagnosis on the basis of a histological examination of the initial breast tumor. ER and PR, which are used as prognostic indicators for breast cancer, can be positive in 32% and 12% of gastric cancer patients, respectively, and are not suitable for differentiating between primary gastric cancer and gastric metastasis of breast cancer (10). GATA-3 positivity is present in 100% of cases of invasive lobular carcinoma of the breast and in 96% of cases of invasive ductal carcinoma of the breast and is considered a marker for breast cancer (9). The positivity rate of GATA-3 in gastric cancer patients is only 5%, making it an important basis for distinguishing between primary and secondary malignant tumors. The comparison of the immunohistochemistry results in this case strongly suggest gastric metastasis.

Recent studies have revealed that the combination of a CDK4/6 inhibitor with hormone therapy is the first choice for the treatment of HR+/HER2- metastatic breast cancer (MBC) because this strategy improves survival (11, 12). Palbociclib, dalpiciclib, ribociclib and abemaciclib are all CDK4/6 inhibitors that are associated with similar survival data for MBC patients. CDK4/6 inhibitors can inhibit the activity of cyclin kinases and block cell proliferation. CDK4/6 inhibitors have changed the treatment paradigm for HR+/HER-2- advanced breast cancer, not only by extending the duration of endocrine therapy but also by delaying the time for patients to receive chemotherapy. The MONARCH-3 study revealed that in postmenopausal HR+/HER-2- advanced breast cancer patients who had not previously received systemic therapy, the median PFS of the abemaciclib group was significantly longer than that of the endocrine therapy group (28.2 months *vs*. 14.8 months), with ORRs of 49.7% and 37%, respectively (13). The DAWNA-1 study results revealed that, compared with that of the control group, the median PFS of the dalpiciclib group was significantly prolonged (15.7 months *vs*. 7.2 months), and the risk of progression or death was decreased by 58% (14). In this case, treatment with dalpiciclib combined with fulvestrant was administered as first-line treatment in the advanced stage, with a PFS of 10 months and good tolerability.

HER-2 overexpression has always been considered a necessary indicator for HER-2 targeted therapy (i.e., IHC3+ or IHC2+/ISH positive). In this case, the initial pathological and recurrent pathological examinations revealed low HER-2 expression, whereas the postoperative pathological examination revealed HER-2 overexpression, which may be related to tumor heterogeneity and may also be related to treatment regimens, examination methods, and judgment criteria. In response to the patient’s postoperative HER-2 overexpression, we administered hormone therapy combined with trastuzumab and pertuzumab as adjuvant treatment, but unfortunately, the patient’s DFS was only 10 months. There is evidence that T-DXd can be used to treat breast cancer patients with low HER-2 expression (15). T-DXd is an antibody–drug conjugate (ADC) composed of the humanized anti-HER-2 monoclonal antibody trastuzumab, the payload topoisomerase I and a tetrapeptide-based cleavable linker. After T-DXd binds to HER-2, it delivers cytotoxic drugs to cancer cells and neighboring cells through the bystander effect. The DESTINY-Breast 4 study (NCT3734029) revealed that regardless of HR status, the progression-free survival (PFS) and overall survival (OS) of the T-DXd treatment group were significantly different. In the HR-positive patient group, the median PFS (mPFS) of the T-DXd group was 4.7 months longer than that of the chemotherapy group (10.1 months *vs*. 5.4 months), with a 49% reduction in the risk of disease progression or death (HR=0.51, 95% CI: 0.4~0.64, P<0.001); in the HR-negative group, T-DXd also had an advantage, with an mPFS of 8.5 months *vs*. 2.9 months (HR=0.46) and an mOS of 18.2 months *vs*. 8.3 months (HR=0.48). The DESTINY-Breast4 study revealed that T-DXd may be a treatment option for advanced breast cancer patients with low HER-2 expression (16). This patient, who was determined to have low HER-2 expression, was given T-DXd as second-line treatment in the advanced stage and is currently under clinical follow-up.

Although breast cancer metastasis to the stomach is not common, the possibility of metastasis should not be overlooked to avoid delaying the diagnosis and treatment of advanced breast cancer. Distinguishing between primary tumors and metastatic tumors is crucial, as doing so directly affects the choice of treatment plan and patient prognosis.

## Data Availability

The raw data supporting the conclusions of this article will be made available by the authors, without undue reservation.
